# Malaria Policy Advisory Committee to the WHO: conclusions and recommendations of September 2012 meeting

**DOI:** 10.1186/1475-2875-11-424

**Published:** 2012-12-19

**Affiliations:** 1 Global Malaria Programme, World Health Organization, 20 Avenue Appia, CH-1211, Geneva 27, Switzerland

**Keywords:** Global, Malaria, WHO, Policy development, Sulphadoxine-pyrimethamine, Pregnancy, Prevention, Primaquine, Gametocytes, *Plasmodium falciparum*, *Plasmodium vivax*, Diagnostic tests, Drug resistance, Surveillance, Mosquito control

## Abstract

The Malaria Policy Advisory Committee to the World Health Organization met in Geneva, Switzerland from 11 to 13 September, 2012. This article provides a summary of the discussions, conclusions and recommendations from that meeting.

Meeting sessions included: updated policy recommendations on the use of sulphadoxine-pyrimethamine for Intermittent Preventive Treatment of malaria in pregnancy, as well as the use of single dose primaquine as a *Plasmodium falciparum* gametocytocide; the need to develop a Global Technical Strategy for Malaria Control and Elimination 2016– 2025 and a global strategy for control of *Plasmodium vivax*; the Affordable Medicines Facility for malaria independent evaluation and promoting malaria case management in the private sector; updates from the Technical Expert Group on drug resistance and containment and the Evidence Review Group on malaria burden estimation; update on the RTS,S/AS01 malaria vaccine; progress on the policy setting process for malaria vector control; and the process for updating the WHO Guidelines for the Treatment of Malaria.

Policy statements, position statements, and guidelines that arise from the MPAC meeting conclusions and recommendations will be formally issued and disseminated to World Health Organization Member States by the World Health Organization Global Malaria Programme.

## Background

The Malaria Policy Advisory Committee (MPAC) to the WHO met from 11 to 13 September 2012 in Geneva, Switzerland, following its inaugural meeting in February 2012 [1]. This article provides a summary of the discussions, conclusions and recommendations from that meeting ^a^ as part of the recently established *Malaria Journal* thematic series “WHO global malaria recommendations” [2].

The following sections of this article provide details and references for the background documents presented at the open meeting sessions of the committee on: the use of sulphadoxine-pyrimethamine for Intermittent Preventive Treatment of malaria in pregnancy (IPTp-SP); the use of single dose primaquine as a *Plasmodium falciparum* gametocytocide; the need for a Global Technical Strategy for Malaria Control and Elimination 2016– 2025 and a global strategy for control of *Plasmodium vivax*; the Affordable Medicines Facility for malaria (AMFm) independent evaluation and promoting malaria case management in the private sector; updates from the Technical Expert Group (TEG) on drug resistance and containment and the Evidence Review Group (ERG) on malaria burden estimation; update on the RTS,S/AS01 malaria vaccine; progress on the policy setting process for malaria vector control; and the process for updating the WHO Guidelines for the Treatment of Malaria.

The MPAC discussion and recommendations related to these topics, which took place partially in closed session, are also included. MPAC decisions are reached by consensus [3]. The next meeting of the MPAC will be 13 to 15 March 2013 [4].

### Report from the WHO Global Malaria Programme

The Director of the WHO Global Malaria Programme (WHO-GMP) updated MPAC members on progress with recommendations from their last meeting [5], including the finalization of the policy recommendation on Seasonal Malaria Chemoprevention (SMC) [6] and on the interim position statement on larviciding in sub-Saharan Africa [7].

Since the previous MPAC meeting, WHO-GMP has launched the *Global Plan for Insecticide Resistance Management in malaria vectors* (GPIRM) [8] on 15 May 2012. In addition, the WHO Director-General launched two new manuals: *Disease Surveillance for Malaria Control* and *Disease Surveillance for Malaria Elimination* in Namibia on World Malaria Day [9]. The Director-General also used that day to launch a new initiative led by WHO-GMP, entitled “T3: Test. Treat. Track”, which calls for every suspected case of malaria to receive a diagnostic test, every confirmed case to be treated with an effective quality-assured medicine, and for the disease to be tracked using a timely and accurate surveillance system [10]. Other important departmental activities discussed included capacity building, Country Malaria Programme Reviews, the development of an Elimination Scenario Planning tool (together with the Clinton Health Access Initiative and the Global Health Group), the launch of a series of Elimination Case Studies (together with the Global Health Group and National Malaria Control Programmes) [11], the revision to the handbook for the management of severe malaria, a new project to provide support to five African countries on scale-up of Integrated Community Case Management (iCCM) [12], and the plans for the World Malaria Report 2012. Also provided were an overview of changes in the global landscape for malaria control and elimination including financial challenges; changes within the Global Fund; the work to gather existing documents into a “malaria investment toolkit”; and a major event, “Malaria 2012”, convened by the Government of Australia to focus on the opportunities and challenges for malaria control and elimination in the Asia-Pacific region [13].

MPAC commended the work of GMP, as well as the ERGs and TEG that had been convened since its inaugural meeting. The next update to MPAC will focus on results from the World Malaria Report 2012, and will include updates from the WHO Regional Offices.

### Intermittent preventive treatment of malaria in pregnancy using sulphadoxine-pyrimethamine (IPTp-SP)

WHO-GMP, at the recommendation of MPAC, convened an ERG on IPTp-SP from 9 to 11 July, 2012 in Geneva [14,15]. The conclusions of the evidence review group [16,17] on the current efficacy and effectiveness of IPTp with SP, which is WHO policy, were evaluated by MPAC. The review indicates that in sub-Saharan Africa, in spite of the increased prevalence in *P. falciparum* of molecular markers associated with resistance to SP (based on the prevalence of quintuple mutant *dhp*s/*dhfr* haplotypes), IPTp-SP remains effective at preventing peripheral parasitaemia, maternal anaemia, and clinical malaria during pregnancy and is associated with reduced neonatal mortality as well as reduced incidence of adverse pregnancy outcomes.

MPAC members agreed with the conclusions of the review, which included new evidence of increased effectiveness of multiple doses of SP (median four) compared to two doses. They endorsed an update of the current recommendation, aiming to improve coverage of and increase access to IPTp with SP as part of routine antenatal care services. The MPAC also recommended that IPTp with SP should still be administered to women in areas of moderate to high malaria transmission in Africa including those where *P. falciparum* parasites carry quintuple mutations linked to SP resistance.

The updated IPTp-SP policy, which will be communicated subsequently to countries by WHO, is as follows [18]:

In areas of moderate-to-high malaria transmission, IPTp with SP is recommended for all pregnant women at each scheduled antenatal care visit, except during the first trimester. WHO recommends a schedule of four antenatal care visits.

a) The first IPTp-SP dose should be administered as early as possible during the 2^nd^ trimester of gestation;

b) Each SP dose should be given at least one month apart;

c) The last dose of IPTp with SP can be administered up to the time of delivery, without safety concerns.

IPTp should ideally be administered as directly observed therapy (DOT); SP can be given either on an empty stomach or with food; folic acid at a daily dose equal or above 5 mg should not be given together with SP as this counteracts its efficacy as an anti-malarial; instead, WHO recommends daily iron and folic acid supplementation in pregnant women at the dose of 30–60 mg of elemental iron and 0.4 mg of folic acid, to reduce the risk of low birth weight infants, maternal anaemia and iron deficiency at term. SP should not be administered to women receiving co-trimoxazole prophylaxis.

MPAC noted that although transmission of malaria has been reduced substantially in some countries where IPTp with SP is currently being implemented, in the absence of information on the level of malaria transmission below which IPTp-SP is no longer cost-effective, such countries should not stop IPTp.

MPAC concluded that monitoring of IPTp-SP effectiveness and safety of multiple doses is essential and should continue. However, research is ongoing to define the best methodology for such monitoring; this will be shared when available. There is currently insufficient evidence to support a general recommendation for the use of IPTp-SP outside Africa.

MPAC suggested that the updated recommendation on IPTp-SP, now finalized and available on the WHO-GMP website [18], should include a brief contextual preamble in addition to the appropriate footnotes. It will subsequently be communicated to relevant countries by WHO-GMP.

### Primaquine single dose as a *P. falciparum* gametocytocide

The ERG on the use of single dose primaquine as a *P. falciparum* gametocytocide [19,20], convened by WHO-GMP at the recommendation of MPAC, presented a summary of their 13 to 15 August, 2012 meeting in Bangkok, Thailand, and the conclusions of their evidence review on the safety and effectiveness of primaquine as a gametocytocide for *P. falciparum*[21,22]. WHO already recommends single dose primaquine (0.75 mg base/kg) for uncomplicated *P. falciparum* malaria, as a component of pre-elimination or elimination programmes, provided that the risks of haemolysis in glucose-6-phosphate dehydrogenase (G6PD) deficient patients are considered.

Although the evidence suggests that primaquine as a gametocytocidal drug can potentially reduce malaria transmissibility, especially in efforts to eliminate *P. falciparum* malaria, the effect on reducing malaria transmission using primaquine requires that a very high proportion of people with *P. falciparum* infection receive it. The concerns related to acute haemolytic anaemia in G6PD-deficient patients and the limited availability of G6PD testing in the field have hampered the successful implementation of this recommendation.

The ERG review suggests that a single 0.25 mg base/kg is effective in reducing transmission and is unlikely to cause serious toxicity in subjects with any of the G6PD variants [21]. How to communicate this clearly – that a lower dose of primaquine is effective and safe and can be administered without G6PD testing – and without causing confusion was the subject of much discussion. MPAC recognized that this recommendation may raise the issue of whether countries already using a single dose of 0.75 mg base/kg primaquine in the treatment of *P. falciparum* malaria (the current WHO recommendation) should consider changing to the lower dose. The conclusion was that these countries should continue with the 0.75 mg base/kg policy until additional information on the efficacy of the lower dose is available, at which time WHO would review the recommendation for these countries.

For countries which had either: (a) areas threatened by artemisinin resistance where single dose primaquine as a gametocytocide for *P. falciparum* malaria is not being implemented; and/or (b) elimination areas which have not yet adopted primaquine as a gametocytocide for *P. falciparum* malaria, MPAC recommended that a single primaquine dose of 0.25 mg base/kg be given to all patients with parasitologically-confirmed *P. falciparum* malaria on the first day of treatment, in addition to an artemisinin-based combination therapy (ACT) drug, except for pregnant women and infants <1 year of age.

The updated recommendation has since been finalized and is available on the WHO-GMP website [23]. It will subsequently be communicated to relevant countries by WHO-GMP. The full evidence review will be published as a WHO publication.

### Developing a global technical strategy for malaria control and elimination 2016– 2025

The Global Malaria Action Plan (GMAP) was launched by the Roll Back Malaria (RBM) Partnership in 2008 following a consultative process with a wide range of stakeholders. While the GMAP does not contain an end date, there has been a request by some members of the RBM Board to consider revising the GMAP before the end of 2015. This will be a point for discussion at the December 2012 RBM Board meeting.

The question posed to MPAC by WHO-GMP for its September session was whether the collection of WHO policy recommendations for malaria is sufficiently clear as they are now, or whether an over-arching review of the strategy mix should be commissioned by WHO-GMP under the oversight of the MPAC [24]. This strategy mix would help underpin any revisions to the GMAP, which would focus on the advocacy, resource mobilization and partner harmonization required to support countries to implement the recommended technical strategy.

MPAC supported the idea that WHO-GMP should develop what it called a Global Technical Strategy for Malaria Control and Elimination, 2016–2025, a period which was perceived as a reasonable and feasible time frame. The concept of stratification and district (peripheral) capacity for malaria control should be central to such an approach. It would also be an opportunity to review a “menu” of options at the country level and consider prioritization, particularly the need for surveillance, monitoring, evaluation, and research. MPAC stressed that it was important to have a bottom-up, country driven approach to the development of this document. It suggested that an ERG be convened to provide the technical input for the intervention mix and stratification that would be central to the new strategy.

MPAC also strongly supported the idea of a revised GMAP that had buy-in from a broad range of stakeholders and sectors. Key suggestions included that it should: (a) be based on a foundation of the WHO Global Malaria Technical Strategy for Malaria Control and Elimination, 2016–2025; (b) address financial and operational elements; (c) be a concise document; (d) RBM and WHO should work closely together in its development; and (e) its goals should be realistic and measurable.

There was consensus from MPAC members and observers that what is needed today is different from what was needed when GMAP was first launched. At that time the focus was on scale-up, and GMAP provided a useful umbrella for this. At present, the new focus should address the heterogeneity and changing dynamics of malaria in order to secure continued progress and in particular, guide countries and regions. MPAC concluded that there was a need for WHO to play a stronger role in providing clear technical strategies to countries, who struggle to reconcile divergent technical guidance, particularly with regard to elimination.

Although MPAC saw developing technical strategies as a core function of WHO-GMP, it advised that any “roadmap to eradication”, currently also under consideration by the global malaria community, would be so far-reaching in its depth and breadth, that it was beyond the capacity of WHO-GMP alone, or any single organization for that matter, to address at this point in time. MPAC advised that any roadmap to eradication be kept separate, but that via its Global Technical Strategy for Malaria Control and Elimination 2016–2025, and through other mechanisms, WHO-GMP should be a critical partner in the process for constructing any detailed roadmap.

### Global strategy for *Plasmodium vivax* control and elimination

A plan to develop a global strategy for *P. vivax* control and elimination was presented to MPAC as a follow up to a discussion from its inaugural meeting [25]. Because *P. vivax* is not perceived to be a major killer compared to *P. falciparum*, it is seldom addressed explicitly in malaria strategies and technical documents, and often features as an add-on to strategies primarily designed for *P. falciparum*.

MPAC agreed with the premise that a global strategy for *P. vivax* malaria was urgently needed, and although it should fall under the umbrella of the overall Global Technical Strategy for Malaria Control and Elimination, 2016–2025, it needed to be commissioned as a separate piece of work to ensure that it is fully developed. However, the depth of work required to pull the strategy together is beyond the scope of a regular ERG. MPAC agreed with the proposed method of work, in particular establishing a small steering committee to shape the work required, and the hiring of a consultant to support the entire process, including facilitating the convening of one or more ERGs as required.

A small steering committee, to be convened by WHO-GMP, will guide the development of a global strategy for *P. vivax* control and elimination and will be convened before the end of 2012; an update will be provided to MPAC at its next meeting.

### Affordable Medicines Facility for malaria (AMFm) and promoting malaria case management in the private sector

Briefings on the AMFm, the future of which was under review by the Global Fund Board at the time, were presented by WHO-GMP [26] and by the Chair of the Global Fund Working Group and RBM Task Force on AMFm [27]. Since the MPAC is not involved with funding decisions for the AMFm, discussion focussed on the broader issue of promoting access to quality care in the private sector. The following is a summary of MPAC recommendations regarding malaria case management in the private sector:

a) Access to affordable and quality assured malaria diagnostic testing, notably Rapid Diagnostic Tests (RDTs), should be an integral part of all initiatives aiming at improving access to ACT in both the private and the public sectors.

b) The primary aim of new global initiatives on malaria case management in the private sector should be a holistic approach to improving the management of febrile children, providing access to malaria diagnostic testing and appropriate treatment for malaria and non-malaria febrile illnesses.

c) The priority for access to subsidized medicines and diagnostics should be given to young children, the group at highest risk.

d) The specific country context should be taken into account in the design and implementation of initiatives aiming at subsidizing medicines and diagnostics, in particular differences in health systems, such as access to health care facilities, proportional role of the private sector in providing care, and availability of community-based health services.

e) In designing new initiatives on malaria case management in the private sector, the increased risk of selection and spread of anti-malarial drug resistance (to both artemisinins and partner medicines) should be considered, and measures put in place to ensure targeting of ACT to confirmed malaria patients.

f) More evidence is required with high quality data in relation to the public health targets of AMFm, especially information on use of co-paid quality assured ACT, to provide informed decisions on the public health value of this initiative.

g) Countries which have been included in the pilot phase of AMFm should be supported during the transition phase, building on the lessons learnt.

h) Further opportunities for closer collaboration and interactions between public and private sectors, some of which emerged in AMFm Phase I, should be further explored especially for peripheral health care settings.

i) New initiatives aimed at improving malaria case management in the private sector should have strong components of education, behaviour change, training and communication to promote wider use of diagnostics and adherence to test results.

j) All future initiatives including subsidies for ACT and RDTs should be designed with careful attention to mechanisms to ensure sustainability.

### Drug resistance and containment

Members [28] of the newly created standing TEG on drug resistance and containment met for the first time from 21 to 22 June 2012, based on the recommendation of MPAC at its inaugural meeting and the subsequent approval of the terms of reference of the TEG [29]. A summary of the meeting and the TEG’s recommendations were presented to MPAC for information and discussion. Updates were presented on monitoring anti-malarial drug efficacy and capacity strengthening, as well as on containment activities, including: a review of the current working definition of artemisinin drug resistance; potential modifications to existing containment strategies; artemisinin resistance outside the Greater Mekong sub-Region; and gaps in research for anti-malarial drug resistance monitoring and containment activities [30,31].

Based on the reviewed data, the TEG concluded that there is currently no evidence of artemisinin resistance outside the Greater Mekong sub-Region; however the TEG recommended continued and intensified surveillance on ACT efficacy outside the Greater Mekong sub-Region and encouraged consultation with the TEG by WHO-GMP whenever new data raise concerns.

MPAC commended Cambodia, which has officially declared elimination as the objective of its anti-malaria programme. In the areas of western Cambodia, which are most affected by artemisinin resistance, the disease is now present in a few foci with low incidence. WHO-GMP will support the Ministry of Health in planning for elimination of *P. falciparum* malaria in these foci within a defined, short time-span.

MPAC supported that mass drug administration be considered as a potentially effective additional measure to achieve elimination rapidly as a containment strategy in such areas. However, pilot studies on mass drug administration for containment of artemisinin resistant malaria, as recommended by WHO following an informal consultation of experts in 2010, have not been implemented yet. MPAC recommended that WHO-GMP should work with National Malaria Control Programmes (NMCPs) to implement one or more pilot studies to assess coverage, effectiveness, safety and other operational issues related to mass drug administration. They also supported the TEG recommendation that one or two NMCP representatives from the Greater Mekong sub-Region be invited to be members of the TEG.

### Malaria burden estimation

Measuring progress in reducing global malaria burden is partially accomplished through the production of estimates of the number of malaria cases and deaths such as those produced by WHO in the World Malaria Report, as well as those reported by other groups. The specific tasks for the ERG on malaria burden estimation, created on the recommendation of MPAC at its inaugural meeting and as outlined in its terms of reference [32], include mapping a way forward in producing malaria burden estimates. The focus was on the use of estimates by WHO Member States and their development partners, as well as describing how to obtain better data for input into those estimates, which can be a challenge [1].

ERG members [33] met for the first of three planned meetings from 27 to 28 June, 2012. As part of its update to MPAC, the ERG Chair described the group’s general timeline, progress to date, and future work [34]. Currently, three meetings are scheduled over approximately 18 months, with supportive work to be done by group members in between. The first meeting [35] was designed to outline relevant issues, the second to elicit further expert opinion, especially from groups directly involved in malaria burden estimation, and the third to develop recommendations to MPAC on the way forward, which will be included in the final meeting report. The objective is to ameliorate the problems with malaria burden estimation in the short term, and to move towards solving them in the long term.

MPAC members acknowledged that the task of the ERG was ambitious, but said that given the weaknesses of current methods, which are most challenging where burden is greatest, consistency and improvements in malaria burden estimation would be invaluable for prioritization and resource allocation. This view was strongly supported by observers present at the meeting, particularly donors, who also suggested that it would be helpful to make clear that as methods have improved and been utilised, previous estimates have been changed retrospectively so that they are comparable.

The issue of how to communicate uncertainty in estimates to politicians, the media, and the public was also discussed. MPAC members stressed that the purpose of the review was not to create a competition among methods, but rather to move in the direction of creating a balance of what methods would be most useful and most feasible at the international level. All members of the global malaria community need to work collaboratively in better communicating uncertainty for any type of estimation to non- malaria specialist audiences.

This ongoing ERG will next update MPAC on its progress at their meeting from 13–15 March 2013.

### RTS,S/AS01 malaria vaccine

RTS,S/AS01, the most advanced vaccine candidate against *P. falciparum*, is currently being evaluated in a pivotal Phase 3 trial. In its update to MPAC [36,37], the Joint Technical Expert Group (JTEG) on Malaria Vaccines [38], first convened in June 2009 by WHO-GMP and the WHO Department of Immunization, Vaccines & Biologicals (WHO-IVB), described RTS,S/AS01 results to date, the timing of further Phase 3 results, the intended target population for deployment, and the timing for any policy recommendations.

The JTEG has determined that there should be sufficient data available to make a draft policy recommendation regarding RTS,S/AS01 in 2015 for subsequent consideration by the policy advisory committees in WHO-IVB (Strategic Advisory Group of Experts -SAGE) and GMP (MPAC). RTS,S/AS01 will be considered as an addition to, not a replacement for, existing preventive and treatment measures. MPAC will have a key role in the decision whether RTS,S/AS01 should be added to the current range of malaria prevention measures and, if so, in which epidemiological situations. SAGE will have a key role for recommendations regarding adding RTS,S/AS01 to routine immunization programmes, its schedule, and ensuring satisfactory co-administration data.

A joint MPAC/SAGE session has been tentatively agreed for April 2015; confirmation depends on results made available to WHO in 2014. Given the apparent waning of vaccine efficacy over time against the first or only episode of clinical malaria, the JTEG highlighted the need for further analyses to explore duration of protection in the full trial results to be received in 2014. The WHO policy recommendation will take into consideration safety and efficacy results from the current Phase 3 efficacy trial after a 30-month follow-up of children who have received the malaria vaccine together with routine infant vaccines, as well as site-specific data on efficacy (where there is adequate power), 18-month booster dose efficacy, and protective efficacy against severe malaria.

### Proposed policy setting mechanisms for malaria vector control

WHO-GMP updated MPAC on progress and challenges in malaria vector control; potential threats and the need for new tools and technologies; and the establishment of a Vector Control Advisory Group (VCAG) to facilitate the review of new paradigms in vector control for both malaria and neglected tropical diseases. WHO-GMP also proposed potential alternatives for advisory mechanisms beyond VCAG to address broader gaps in policies for malaria vector control [39,40], in particular the technical guidance for malaria vector control programmes since VCAG, as a non-malaria specific body, is not designed to address the full range of malaria vector control policy issues.

After being asked to consider whether to establish a standing TEG for malaria vector control or to convene time-limited ERGs as the need for specific policy decisions arises, and following clarifications regarding the roles of the various malaria vector control groups, MPAC recommended that there is a need for a standing TEG; ERGs may also be convened as needed.

Following MPAC inputs on the structure of the TEG and a review of existing TEGs [41], terms of reference have since been finalized and are available on the WHO-GMP website. A call for expressions of interest in TEG membership will be disseminated by WHO-GMP.

### Chemotherapy TEG and process for updating WHO Guidelines for the Treatment of Malaria

The terms of reference for the TEG on malaria chemotherapy, a group that pre-dates the creation of MPAC, were presented to MPAC for approval [42] in order to be in line with the new structure and process for global malaria policy setting. They were agreed and have been published on the WHO-GMP TEG webpage [43]. After extensive discussion, there was agreement that the TEG should remain a single group, encompassing both treatment and chemoprevention. A proposal for rotating membership was also discussed and agreed, so that there will be overlap between current and new members, particularly in light of the soon-to-be-updated WHO Guidelines for the Treatment of Malaria, which will be a significant part of the TEG’s work in 2013.

The full process for reviewing and updating the WHO Guidelines for the Treatment of Malaria were presented and discussed in detail [44,45]. Several suggestions were made to improve the format of what will be the third edition of the Guidelines, including adding clear summaries for policy makers, and increasing the number of languages in which the guidelines are published (currently English, French, and Spanish). WHO-GMP welcomed any further feedback or suggestions from the global malaria community that would improve the third edition of these Guidelines.

## Discussion

The wording for policy recommendations were finalized by MPAC during their closed session and via email following the meeting; conclusions have been included in the summaries of the meeting sessions above, and links to the full recommendations have been provided as references. Depending on the agenda for the next MPAC meeting, time allocated to the closed session might be increased to maximize the benefit of having MPAC members together to finalize recommendations.

Position statements and policy recommendations made by the MPAC are approved by the WHO Director-General, and will be formally issued and disseminated to WHO Member States by WHO-GMP. Conclusions and recommendations from MPAC meetings are published in the *Malaria Journal* as part of this series.

MPAC will provide suggestions for the agenda for its next meeting to the WHO-GMP Secretariat. Feedback will also be given to and received from the global malaria community at the RBM Board meeting in December 2012, and through the publication of, and correspondence, regarding this article.

On-going engagement with and attendance by interested stakeholders at MPAC meetings was strongly encouraged; MPAC members were pleased by the diversity and increase in the number of observers in attendance at the September meeting [46] and encouraged WHO-GMP to continue its outreach. In addition to open registration for MPAC meetings, which will continue, and attendance by four standing observers (RBM, the Global Fund, UNICEF, Office of the UN Special Envoy for malaria), the active participation of seven rotating NMCP representatives, and all six WHO Regional Malaria Advisors, at the September 2012 and future meetings was strongly welcomed.

## Conclusion

The meeting feedback received from participants and observers, and MPAC members themselves, was very positive. Having only met twice to date, MPAC is still in the process of orienting itself to best serve the needs of the global malaria community. However, the format of MPAC meetings and its feedback loops with other advisory bodies and stakeholders is beginning to take shape. WHO-GMP and the MPAC continue to strongly welcome feedback, support, and suggestions for improvement to MPAC meetings from the global malaria community.

The next meeting of the MPAC will take place from 13 to 15 March 2013 in Geneva, Switzerland. Further information including the agenda and details on how to register will be made available in early 2013 on the WHO-GMP website for MPAC [4].

## Endnotes

^a^The complete set of all MPAC September 2012 meeting-related documents including background papers, presentations, and member declarations of interest can be found online at http://www.who.int/malaria/mpac/sep2012/en/index.html.

## Abbreviations

MPAC: Malaria Policy Advisory Committee; IPTp-SP: Intermittent Preventive Treatment of malaria in pregnancy using sulphadoxine-pyrimethamine; AMFm: Affordable Medicines Facility for malaria; TEG: Technical Expert Group; ERG: Evidence Review Group; WHO-GMP: World Health Organization Global Malaria Programme; SMC: Seasonal Malaria Chemoprevention; GPIRM: Global Plan for Insecticide Resistance Management in malaria vectors; iCCM: Integrated Community Case Management; DOT: Directly Observed Therapy; G6PD: Glucose-6-phosphate dehydrogenase; ACT: Artemisinin-based combination therapy; GMAP: Global Malaria Action Plan; RBM: Roll Back Malaria; RDT: Rapid Diagnostic Test; NMCP: National Malaria Control Programme; JTEG: Joint Technical Expert Group; WHO-IVB: WHO Department of Immunization, Vaccines & Biologicals; SAGE: Strategic Advisory Group of Experts; VCAG: Vector Control Advisory Group.

## Competing interests

The authors declare that they have no competing interests.

## Authors’ contribution

All authors listed below have equally contributed to the article. All authors have read and approved the final version of the manuscript.

## Authors’ information

WHO Malaria Policy Advisory Committee Members

· Salim Abdulla, Ifakara Health Institute, Dar Es Salaam, United Republic of Tanzania.

· Pedro Alonso, Centre for International Health and Research, Barcelona, Spain.

· Fred Binka, University of Ghana, Accra, Ghana.

· Patricia Graves, James Cook University, Cairns, Australia.

· Brian Greenwood, London School of Hygiene and Tropical Medicine, London, United Kingdom.

· Rose Leke, University of Yaoundé, Yaoundé, Cameroon.

· Elfatih Malik, Ministry of Health, Gezira, Sudan.

· Kevin Marsh, Kenya Medical Research Institute, Kilifi, Kenya.

· Sylvia Meek, Malaria Consortium, London, United Kingdom.

· Kamini Mendis, Columbo, Sri Lanka.

· Allan Schapira, Legazpi City, Philippines.

· Laurence Slutsker, Centers for Disease Control and Prevention, Atlanta, United States of America.

· Marcel Tanner, Swiss Tropical Public Health Institute, Basel, Switzerland.

· Neena Valecha, National Institute of Malaria Research, New Delhi, India.

· Nicholas White, Mahidol University, Bangkok, Thailand.

WHO Malaria Policy Advisory Committee Secretariat

· Andrea Bosman, WHO Global Malaria Programme, Geneva, Switzerland.

· Richard Cibulskis, WHO Global Malaria Programme, Geneva, Switzerland.

· Bianca D'Souza, WHO Global Malaria Programme, Geneva, Switzerland and London School of Hygiene and Tropical Medicine, London, United Kingdom.

· Abraham Mnzava, WHO Global Malaria Programme, Geneva, Switzerland.

· Vasee Moorthy, WHO Immunization, Vaccines and Biologicals Department, Geneva, Switzerland.

· Robert Newman, WHO Global Malaria Programme, Geneva, Switzerland.

· Peter Olumese, WHO Global Malaria Programme, Geneva, Switzerland.

· Pascal Ringwald, WHO Global Malaria Programme, Geneva, Switzerland.
